# TRPV6 alleles do not influence prostate cancer progression

**DOI:** 10.1186/1471-2407-9-380

**Published:** 2009-10-26

**Authors:** Thorsten Kessler, Ulrich Wissenbach, Rainer Grobholz, Veit Flockerzi

**Affiliations:** 1 Institut für Experimentelle und Klinische Pharmakologie und Toxikologie, Universität des Saarlandes, Homburg/Saar, Germany; 2 Institut für Allgemeine und Spezielle Pathologie, Universität des Saarlandes, Homburg/Saar, Germany

## Abstract

**Background:**

The transient receptor potential, subfamily V, member 6 (TRPV6) is a Ca^2+ ^selective cation channel. Several studies have shown that TRPV6 transcripts are expressed in locally advanced prostatic adenocarcinoma, metastatic and androgen-insensitive prostatic lesions but are undetectable in healthy prostate tissue and benign prostatic hyperplasia. Two allelic variants of the human *trpv6 *gene have been identified which are transcribed into two independent mRNAs, TRPV6a and TRPV6b. We now asked, whether the *trpv6a *allele is correlated with the onset of prostate cancer, with the Gleason score and the tumour stage.

**Methods:**

Genomic DNA of prostate cancer patients and control individuals was isolated from resections of prostatic adenocarcinomas and salivary fluid respectively. Genotyping of SNPs of the TRPV6 gene was performed by restriction length polymorphism or by sequencing analysis. RNA used for RT-PCR was isolated from prostate tissue. Data sets were analyzed by Chi-Square test.

**Results:**

We first characterized in detail the five polymorphisms present in the protein coding exons of the *trpv6 *gene and show that these polymorphisms are coupled and are underlying the TRPV6a and the TRPV6b variants. Next we analysed the frequencies of the two TRPV6 alleles using genomic DNA from saliva samples of 169 healthy individuals. The homozygous TRPV6b genotype predominated with 86%, whereas no homozygous TRPV6a carriers could be identified. The International HapMap Project identified a similar frequency for an Utah based population whereas in an African population the a-genotype prevailed. The incidence of prostate cancer is several times higher in African populations than in non-African and we then investigated the TRPV6a/b frequencies in 141 samples of prostatic adenocarcinoma. The TRPV6b allele was found in 87% of the samples without correlation with Gleason score and tumour stage.

**Conclusion:**

Our results show that the frequencies of *trpv6 *alleles in healthy control individuals and prostate cancer patients are not significantly different. Although expression of *trpv6 *transcripts correlates with aggressive potential of prostate cancer, the TRPV6 genotype does not correlate with the onset of prostate cancer, with the Gleason score and the tumour stage.

## Background

Intracellular calcium levels are highly regulated to achieve precise regulation of cell signalling pathways. These pathways lead to muscle contraction, synaptic transmission, hormone secretion as well as apoptosis and cell proliferation. The major protein classes involved in calcium homeostatsis are calcium pumps, sodium/calcium exchangers and Ca^2+ ^permeable cation channels. An upregulation of IP_3 _receptor channels [1,2], voltage gated calcium channels [3-5] and TRP-channels has been demonstrated in various cancer tissues including prostatic adenocarcinoma tissue [6-8]. Among the TRP-calcium channel family TRPM4 has been shown to be expressed in benign and malign prostate tissue [9]whereas TRPM8 is overexpressed in prostate cancer [6]. TRPM8 and TRPM4 are non-selective cation channels and TRPM4 is not permeable for Ca^2+^. In contrast TRPV6 is a highly Ca-selective cation channel and its transcripts are overexpressed in prostatic adenocarcinoma but undetectable in healthy and benign prostate tissue [7,10]. In prostate tissue the expression of TRPV6 is strictly correlated with the Gleason grading and the tumour staging implying that TRPV6 is an indicator for the metastatic potential of prostatic adenocarcinoma and a potential target for drugs which may be used to treat this disease [11]. Analysis of 40 tissue samples showed that TRPV6 transcripts occur in more than 90% of patients with extraprostatic prostatic adenocarcinoma indicating that patients with TRPV6 positive tumours have a bad prognosis [11]. Overexpression of TRPV6 stimulates proliferation of HEK293 cells in a calcium dependent manner [12].

Previously we isolated two allelic TRPV6 variants from human placenta [7]. The cDNAs of these variants differ in 5 base pairs (bp). Two nucleotide substitutions, a1080g and g1787a, are silent whereas three substitutions resulted in changes of the encoded amino acid residues R_157 _to C_157 _(R157C), V_378 _to M_378 _(V378M) and T_681 _to M_681 _(T681M). The resulting protein sequences were called TRPV6a (accession number: AJ243500) and TRPV6b (AJ243501). We suggested that these 5 bp represent a coupled polymorphism [7].

Based on this study we now examined whether genotyping of one of the polymorphisms within the protein coding region of the TRPV6 cDNA is sufficient to predict expression of the TRPV6a or TRPV6b variant. In addition, we asked whether expressing of the TRPV6a or TRPV6b variant, respectively, correlates with the occurrence of prostatic adenocarcinoma, its onset and aggressiveness. We determined the frequencies of the two TRPV6 alleles within Caucasians and compared these frequencies with genotypes obtained from prostatic adenocarcinoma specimen. Apparently, the frequency of either TRPV6 variant is not associated with the onset of prostate cancer and does not correlate to the Gleason score and to the tumour stage.

## Methods

All experiments were approved by the local state's ethical committee (Ethik-Kommission of the Ärztekanmmer des Saarlandes, Saarbrücken, Germany).

### Genotyping of TRPV6 alleles

**Figure 1:**
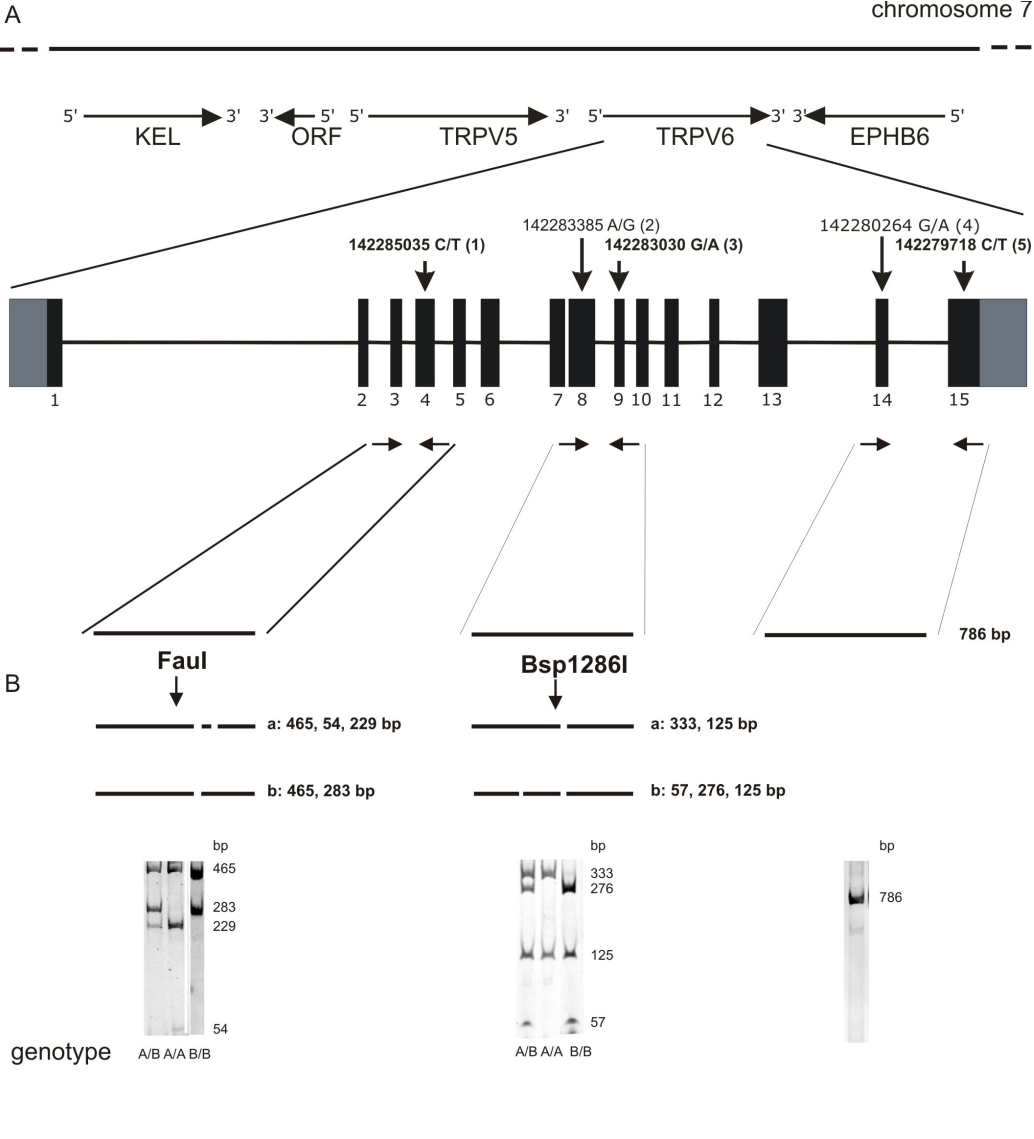
**Genotyping of TRPV6 alleles**. A, TRPV6 locus on human chromosome 7. Genes adjacent to TRPV6 are EPHB (ephrin receptor precursor), TRPV5 and KEL (Kell blood group antigen). Below the structure of the TRPV6 gene with the 15 exons and the coupled polymorphisms 1, 2, 3, 4, and 5. Polymorphisms within introns are not included. The polymorphisms 1, 3 and 5, but not polymorphisms 2 and 4, affect the TRPV6 protein sequence. B, Genotyping of TRPV6 alleles by restriction of amplified DNA using FauI and Bsp1286I to analyze polymorphism 1 (left) and polymorphism 2 (middle) and direct sequencing of amplified DNA to analyze polymorphism 4 and 5 (right). Arrows indicate positions of primer pairs used for DNA amplification.

To analyze polymorphism 1 a genomic DNA fragment of the TRPV6 gene was amplified (primer pair 430/431; see Figure 1 and Table 1) and restricted by FauI; the fragments were separated by 5% polyacrylamide gels. The expected sizes of the fragments were 283 and 465 bp (b/b genotype), 54, 229 and 465 bp (a/a genotype) and 54, 229, 283 and 465 bp (a/b genotype). To analyze polymorphism 2 a genomic DNA fragment of the TRPV6 gene was amplified (primer pair 243/244; see Figure 1 and Table 1) and restricted by Bsp1286I; the expected sizes of the fragments were 57, 125 and 276 bp (b/b genotype), 125 and 333 bp (a/a genotype) and 57, 125, 276 and 333 bp (a/b genotype). To analyze the fourth and fives polymorphism we amplified a genomic fragment of the TRPV6 gene with the primer pair 778/779 and sucloned the amplified DNA in the plasmid pBluescript (Stratagen) and sequenced more than 10 individual clones on both strands.

**Table 1:** Primer used for genotyping

Sequence (5'-3'):
243 caccatgtgctgcatctacc

244 caatgacagtcaccagctcc

430 atggactctgagctctatgagg

431 cccacatctcagctcagg

778 cacggtgaatgctggagcg

779 ccaggaagcgaagtgagaac

780 cgtctgaagcgcacgtcc

781 cttgaagtccgccagcagg

637 gctcgagatgtcatgaagg

638 agttgagagatcatctccacc

### Isolation of genomic DNA and RNA

Genomic DNA was isolated from frozen prostate tissue (~0.5 g) with the QIAamp DNA Mini Kit (Qiagen). Salivary was obtained from healthy individuals of Caucasian origin. Male individuals were without known prostate disease. Genomic DNA from control samples was isolated from salivary using the QIAamp Blood Mini Kit (Qiagen) with the following modifications: The salivary (1 ml) was diluted in 4 ml PBS-buffer, and centrifuged 10 min at 3000 g. The cell sediment was resuspended in 180 μl PBS and digested in the presence of 20 μl ProteinaseK (60 U/ml) and 200 μl AL buffer (Qiagen) at 56°C for 10 min followed by the addition of 200 μl ETOH (100%). The sample was applied to a Qiamp spin column and the genomic DNA was isolated according to the manufacturer instructions. Total RNA was isolated from 0.5 g frozen prostate tissue using the peqGold RNA Pure (Peqlab). First strand was synthesized using the SuperScript First-Strand Synthesis System for RT-PCR (Invitrogen). DNA-sequencing was carried out with a capillary sequencer (ABI) using the BigDye Terminator v3.1 Cycle Sequencing Kit (Applied Biosystems). DNA fragments were subcloned in pBluescript and transformed in XL1 competent cells (Stratagene). Plasmids were isolated using a plasmid Midi Kit (Qiagen). PCR reactions were typically run in a total volume of 50 μl using the phusion proof reading polymerase (NEB). The annealing temperatures were adjusted to 58°C, elongation time depending on fragment length was adjusted to 20 sec/1 kb.

Prostate tissue samples: Patients' informed consent was obtained prior to all investigations. Only patients with primary prostatic acinar adenocarcinomas were included in the study. Radical prostatectomy specimens were placed on ice after removal and immediately transferred to the Institute of Pathology. The native prostate was subsequently processed according standard protocols [13]. Tumour-suspicious and tumour-free areas were resected and snap frozen in liquid nitrogen and stored at -80°C until use. The remaining material was fixed in 4% buffered formaldehyde for 24 hours. The frozen sections of the native material identified tumour and tumour-free areas and were included in the diagnostic process. The grading was performed in accordance to the 2005 modified Gleason score [14]. Low-grade tumours were defined as tumours with Gleason scores 5 to 6, high grade tumours as Gleason scores 7 to 10. The staging was performed in accordance to the 6^th ^edition of the UICC TNM staging system of malignant tumours (2002). Saliva for genotyping was obtained from 169 healthy volunteers. Volunteers informed consent was obtained before all investigations.

Allele frequencies were analyzed by Chi Square test.

## Results

### Coupled polymorphisms of the TRPV6 gene

The human TRPV6 gene is located on the chromosome 7q33-34 adjacent to the TRPV5 and EPHB6 genes and its coding sequence encompasses 15 exons (Figure 1A, [15]). Earlier we isolated TRPV6 cDNA clones from a human placenta cDNA library and found two cDNAs comprising 5 single nucleotide polymorphisms (SNPs, [7]). These polymorphisms are located in exons 4, 8, 9 14 and 15 and were numbered from 1 to 5 (Figure 1). The polymorphisms on exon 8 (a/g) and on exon 14 (g/a) represent silent base pair exchanges whereas changes of c to t (exon 4), g to a (exon 9) and c to t (exon 14) lead to the amino acid substitutions Arg157Cys, Val378Met and Thr681Met in the deduced protein sequence. From the data we concluded that from the two alleles two TRPV6 proteins are translated, one, TRPV6a, with the amino acids R_157_, V_378 _and T_681 _and a second variant, TRPV6b, with the amino acid residues C_157_, M_378 _an M_681_.

The polymorphism on exon 4 can be analyzed by the restriction enzyme FauI, because the sequence of the TRPV6a allele contains an additional FauI restriction side not present in the TRPV6b allele (Figure 1). A second restriction fragment length polymorphism occurs on exon 8 with the restriction enzyme Bsp1286I. To study if the polymorphisms are typically coupled we isolated genomic DNA from salivary obtained from 36 healthy volunteers and amplified DNA fragments using the primer pair 430/431 that flank exons 3 and 4. The amplified DNA fragment covering exons 3 and 4 and the intron sequence in between was restricted with FauI and the fragment length polymorphisms analyzed by polyacrylamide gels. The expected fragment sizes were 465, 229 and 54 bp for TRPV6 alleles a/a, 465, 283, 229, and 54 bp for alleles a/b and 465 and 283, for alleles b/b (Figure 1). Next we analyzed the first polymorphism of the 36 individuals. 19 of the 36 samples were of the b/b genotype and 17 were heterozygous (a/b); the a/a genotype was not detected. To test if this first polymorphism is coupled to the second we amplified the DNA encoding exon 8 and 9, including the intron in between, with the primers 243/244 from the 36 individuals and cut the fragments by Bsp1286I. The 19 samples that exhibited the b/b genotype at the first polymorphic side were b/b genotyped at the second side and as expected the remaining 17 individuals exhibited the heterozygous genotype a/b supporting the conclusion that the polymorphisms within the homozygous individuals, and apparently in the heterozygous individuals are coupled, as suggested previously [7].

TRPV6 is overexpressed in prostatic adenocarcinoma tissue and we next extracted genomic DNA from a total of 142 prostate tissue samples and analyzed the second polymorphism by the restriction enzyme Bsp1286I. The DNA of one out of the 142 tissue samples (sample 88, see Additional file 1) was of the a/a genotype at the second polymorphic side. We analyzed the first polymorphic side and found the genotype a/a indicating that this sample contains homozygous TRPV6a alleles (Figure 1). To test if the fourth and fifth polymorphisms were coupled to the first and second, we further amplified DNA fragments from sample 88 containing exons 14 and 15 using the primer pair 778/779. The amplified DNA fragments were subcloned and ten individual clones were sequenced. The sequence of all a-alleles show that the polymorphisms are coupled. Four prostate tissue samples exhibiting the b/b genotype at the second polymorphic side were analyzed in a similar way. The 4 DNAs were genotype b/b at the first, fourth and fifth polymorphic side. Furthermore we extracted total RNA from a pool of ten prostate cancer patients and amplified the full length coding sequence of the TRPV6 cDNA. The cDNAs of ten individual clones were subcloned and sequenced. Nine clones reflect the TRPV6b and one the a-allele also indicating a coupling of the polymorphisms.

### Allele frequencies of prostate cancer patients

**Figure 2:**
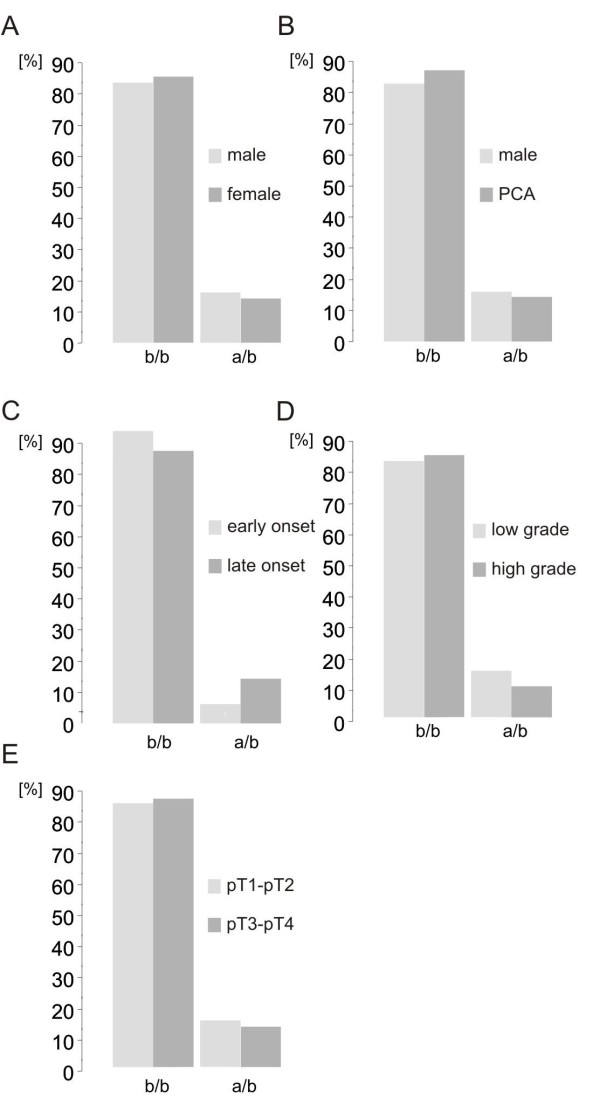
**TRPV6 allele frequencies in prostate cancer patients**. (A) Comparison of TRPV6 allele frequencies in salivary from Caucasian males and females, (n = 169, χ^2 ^= 0.22, p > 0.1), (B) tissue samples from healthy males (n = 77) and prostate cancer patients (n = 141, χ^2 ^= 0.23, p > 0.1), (C) tissue samples from early onset prostate cancer patients (age < 60) and late onset patients (age > 60) (n = 99, χ^2 ^= 0.58, p > 0.1), (D) tissue samples from patients with low Gleason scores (<7) and high Gleason scores (>7) (n = 90, χ^2 ^= 0.69, p > 0.1) and (E) tissue samples from patients with the tumour stages pT1-pT2 and patients with the tumour stages pT3-pT4 (n = 99, χ^2 ^= 0.03, p > 0.1).

The incidence of prostatic adenocarcinoma depends on non genetic factors but also on ethnicity and familial background [16]. We therefore asked if the TRPV6 genotype is correlated with prostatic adenocarcinoma and its progression. We first determined the TRPV6 allele frequencies of 169 healthy Caucasians by analyzing the second polymorphic side in DNA obtained from salivary as described above (Figure 1). We found that 145 (86%) of healthy control individuals were of the homozygous TRPV6 genotype b/b whereas the homozygous genotype a/a was not detected. The allele frequencies of males and females are not statistically different (males 65 (84%); females 80 (87%) (Figure 2A). Data from the HapMap project http://www.HapMap.org/ indicate that the frequency of TRPV6a alleles in Africans is higher [17]. The HapMap project sequenced the genomic locus of TRPV6 of 30 individuals from Yoruba/Nigeria and found a higher frequency of a-alleles within this population (43% a/a, 42% a/b, 15% b/b). Similar TRPV6 allele frequencies in Africans and black Americans were published recently [18,19]. In addition prostatic adenocarcinomas occur more often in the black population of the US [16].

This finding raised the question if a higher TRPV6a allele frequency correlates with an increase of the incidence of prostatic adenocarcinoma. Therefore we compared the TRPV6 allele frequencies from Caucasian control individuals with the DNA obtained from prostatic adenocarcinoma tissue samples. We isolated genomic DNA from 142 prostatic adenocarcinoma resections and genotyped the samples using the restriction enzyme Bsp1286I. 125 (88%) of the prostate tissue samples exhibit the genotype b/b, 16 (11.3%) the heterozegous genotype and only one sample was homozygous a/a (0.7%). These data show that allele frequencies of healthy male individuals (84% b/b) and prostate cancer patients (88% b/b) are very similar (Figure 2B). Prostate cancer patients can be assigned to two cohorts [20], one representing patients below 60 years of age (early onset of prostate cancer) and a second with patients above 60 years (late onset of prostate cancer). Only from 99 of the 142 tissue samples the date when prostatic adenocarcinoma was diagnosed was known. After assigning the tissue samples to either cohort we found allele frequencies of 94% (b/b) and 6% (a/b) in the first and 87% (b/b), 12% (a/b) and 1% (a/a) in the second. Because early onset of prostate cancer is relatively rare only 17 tissue samples of 99 from the patients examined belong to this group. However, the allele frequencies between these groups are similar (Figure 2C) indicating that onset of prostate cancer is independent of the TRPV6 genotype.

Next, we assigned the prostatic adenocarcinoma tissue samples to the Gleason score which describes the differentiation of the prostate tissue and is indicative of the malignancy of a tumour.

The Gleason score is written as the sum of the two most prominent Gleason patterns. A Gleason score of >7 is correlated with a highly malignant tumour whereas a low Gleason score (<7) is an indicator of weaker aggressiveness [21,22].

Similar TRPV6 allele frequencies were found if the prostate tissue samples were grouped according to the Gleason score, 83% of the low grade Gleason tumours exhibited the homozygous b/b genotype, 89% b/b of the high grade Gleason tumours (Figure 2D).

Prostate tumours of stage pT2 are confined to the prostate whereas the tumours of the stages pT3a, pT3b and pT4 are not restricted to the prostate and represent extraprostatic tumours at the time of resection. The tumour stage pT1a/b represents tumours that are found incidentally during the resection of hyperplastic prostate tissue. More than 90% of pT3b tumours express TRPV6 transcripts, whereas TRPV6 expression is only in 20% of pT2 tumours detectable [11]. The percentage of pT1a/b and pT2 tumours which have the potency to develop biological aggressive potential is unknown. It is commonly believed that the fraction of clinical irrelevant tumours within the group of pT1a/b and pT2 tumours is relatively high [23]. Therefore we asked whether TRPV6 allele frequencies of extraprostatic and intra-prostatic tumours (at time of resection) are different or not. Comparing the allele frequencies of patients with pT1a/b and pT2 tumours with patients with extraprostatic tumours (pT3-pT4) we found no significant difference between these two groups. 94% of the pT1a/b and pT2 tumours were genotyped b/b and 87% of the pT3-pT4 tumours (Figure 2E). The results indicate that the TRPV6 genotype is not correlated with an extraprostatic tumour growth. To demonstrate that TRPV6 is indeed expressed in the tumour tissue that we used for genotyping we randomly tested TRPV6 expression by conventional RT-PCR using tumour samples of patients 3, 19, 20, 27 and 87. In all samples TRPV6 specific amplicons were obtained as well as TRPM4 and hypoxanthin phosphoribosyltransferase type 1 (HPRT1) transcripts which we used as positive controls (data not shown).

## Discussion

We show that the 5 polymorphisms of the TRPV6 cDNA that define TRPV6a and TRPV6b alleles are typically coupled. We used the second polymorphisms for genotyping to determine the TRPV6 allele frequencies in healthy Caucasians. We can demonstrate that within the Caucasian population TRPV6b alleles are more frequent than TRPV6a. Because TRPV6 transcripts are present in advanced prostatic adenocarcinoma samples we asked if the TRPV6 genotype may influence the progression of prostate cancer. We genotyped tissue samples from 142 prostate cancer patients and found a similar distribution of TRPV6 alleles as in healthy Caucasians. We show that the TRPV6 genotype is not correlated with the onset of prostate cancer. The small number of probes reflects that early onset of prostate cancer is a rare event. However the data show a tendency that the frequency of TRPV6a alleles in the patient group is lower than in controls. Furthermore the TRPV6 genotype is not correlated with the Gleason score and the tumour stage of prostatic adenocarcinoma samples.

TRPV6 exhibits a highly unusual coupled polymorphism within the human population: While the ancestral allele TRPV6a (R_157_, V_378 _and T_681_) is common among African populations, the derived allele TRPV6b (C_157_, M_378 _and M_681_) is predominant in all other tested population groups suggesting that this haplotype conferred a temporally or geographically selective advantage [19]. The reason for this selection has remained elusive and no obvious differences in ion channel properties have been found [24]. In addition, the incidence of prostatic adenocarcinoma within African/Afro-American populations is higher than in Caucasians [25-27]. Previously we have shown that TRPV6 overexpression correlates with extraprostatic prostatic adenocarcinoma [11] and we asked whether the ancestral TRPV6a allele is associated with this finding. Our findings do not support the assumption that the TRPV6a allele is correlated with a higher incidence of prostatic adenocarcinoma.

We found only one patient with the genotype a/a and we cannot exclude at this stage that the homozygous TRPV6a allele is associated with the higher prostatic adenocarcinoma incidence found in African/Afro-American people.

We re-analyzed the polymorphism of the TRPV6 locus which had been identified by the HapMap project. The HapMap project sequenced the genomic TRPV6 locus of four ethnic groups (Yoruba/Nigeria, Utah/USA-European based, Tokio/Japan, Beijing/China). These data include the second polymorphism that we analyzed by RFLP and the resulting genotype frequencies are summarized in Table 2. It is striking that depending on the distance to the African continent the frequency of the homozygous TRPV6 b/b genotype increases and it was postulated that the TRPV6 locus may be a target to positive selection thus leading to an enhanced b/b genotype frequency within the Europeans, European based Americans and within Asian populations [18,28].

**Table 2:** TRPV6 allele frequencies of four ethnic groups compared with data from this study

TRPV6 genotype (in %)	a/a	a/b	b/b
Yorube (Africa)	43	42	15
Utah (USA)	0	12	88
Tokyo (Japan)	0	5	95
Beijing (China)	0.3	12.7	87

Data taken from the International HapMap Project (http://www.HapMap.org/; [17]. The International Hap Map Project Website).

**Figure 3:**
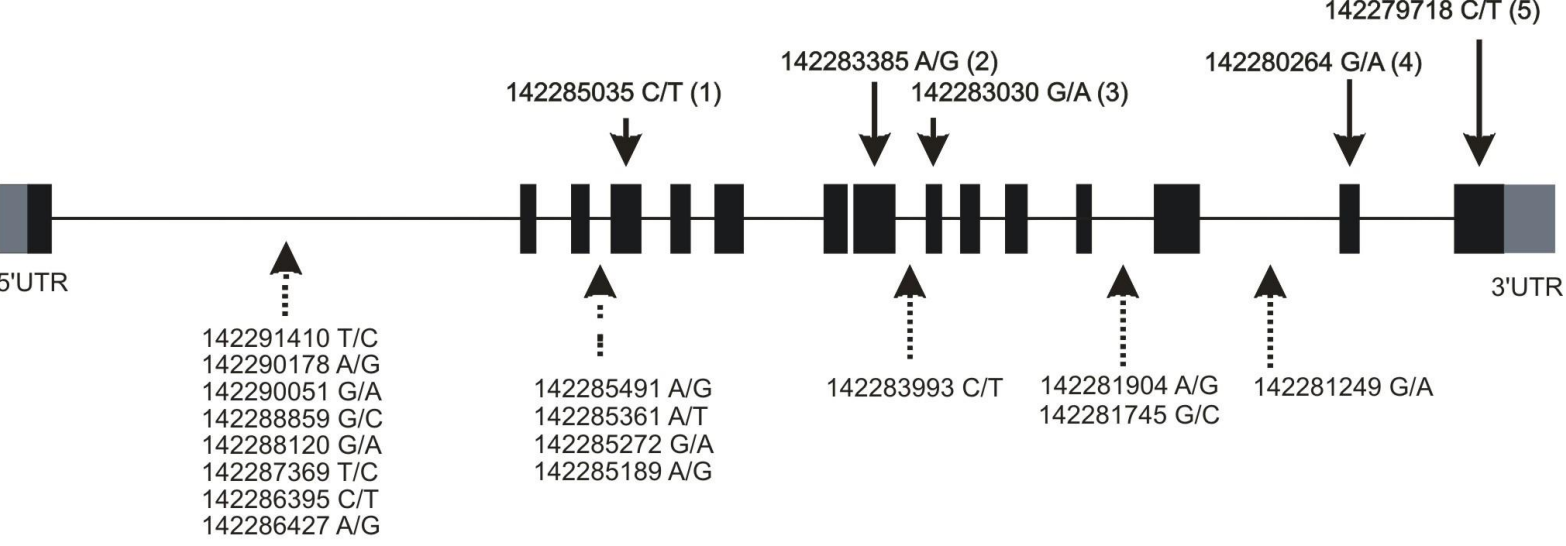
**Polymorphism of TRPV6a and TRPV6b alleles**. The TRPV6 gene consists of 15 exons (black boxes). The five polymorphisms located within protein coding exons are shown on top, polymorphisms within introns are shown below. The polymorphisms within introns http://www.HapMap.org/ are found with similar frequencies as the polymorphisms found within exons (see text).

The TRPV6 locus contains more than 50 polymorphisms which can be found in the HapMap Project (http://www.HapMap.org/, Figure 3). The HapMap data show that the 5 exon derived polymorphisms that we describe and which are occurring in exon coding sequences do have a similar frequency as 16 intron based polymorphisms. In addition these 16 polymorphisms had a similar frequency in the four ethnic groups shown in Table 2 as the 5 polymorphisms found in exon coding sequences described here. Within the most adjacent genes TRPV5, KEL and EPHB6, we did not find polymorphisms with similar frequencies. We speculate that selection pressure leading to an increase of TRPV6b alleles in non-African based populations is based on the TRPV6 locus itself but not on the adjacent genes.

## Conclusion

TRPV6 transcripts are expressed in patients with advanced prostatic adenocarcinoma but are not detectable in healthy and benign prostate tissue. [7]. Within Caucasians two allelic variants, TRPV6a and TRPV6b, are typically present whereas ~87% of this population exhibit the homozygous TRPV6b genotype. In African based Americans TRPV6a alleles are much more frequent. The prostatic adenocarcinoma incidence within African Americans is 2-3 times increased compared to Caucasians. Because Caucasians typically exhibit the homozygous TRPV6b (b/b) or the hetereozygous a/b genotype we asked if the occurrence of the heterozygous genotype in Caucasians is correlated with the Gleason score, the tumour stage or the onset of prostate cancer. From the data we conclude that the TRPV6 genotype does not correlate with the progression of prostatic adenocarcinoma.

## Competing interests

The authors declare that they have no competing interests.

## Authors' contributions

TK carried out the genotyping experiments. RG analysed the prostate tissues, UW identified the polymorphism of the TRPV6 gene, designed the genotyping experiments and analyzed SNP data from the International HapMap Project. UW and VF drafted the manuscript. All authors read and approved the final manuscript.

## Pre-publication history

The pre-publication history for this paper can be accessed here:

http://www.biomedcentral.com/1471-2407/9/380/prepub

## Supplementary Material

Additional file 1 **Classification of prostate cancer samples**. The data provided describe the tumour stage and the Gleason grade of the tumour samples that were used in the study. In addition the age of the patients at time of resection is given and the TRPV6 genotype is indicated. Click here for file
