# Supplementary material for: TRPV6 alleles do not influence prostate cancer progression
Source: BMC Cancer. 2009 Oct 26;9:380. doi: 10.1186/1471-2407-9-380 (PMC2774862; doi:10.1186/1471-2407-9-380)
Supplement: Additional file 1 — Classification of prostate cancer samples. The data provided describe the tumour stage and the Gleason grade of the tumour samples that were used in the study. In addition the age of the patients at time of resection is given and the TRPV6 genotype is indicated. [file 1471-2407-9-380-S1.DOC]

**Additional file 1**

**Classification of prostate cancer samples**

Altogether 142 tissue samples from prostate cancer were available; 100 samples were classified as indicated below. From sample 89 no DNA could be extracted. 42 samples were not classified regarding Gleason score, tumour stage and age of patient. n.a. not available, pT-stage indicates the classification according to the UICC TNM staging system of malignant tumours, 6^th^ edition (2002).

| **Sample** | **Age at operation** | **T(NM)** | **Gleason** | **Genotype** |
| --- | --- | --- | --- | --- |
| PCA |  |  | Score |  |
| 1 | 64 | pT2c | 6 (3+3) | B/B |
| 2 | 64 | pT3b | 7 (4+3) | B/B |
| 3 | 67 | pT2c | 7 (3+4) | B/B |
| 4 | 64 | pT2c | 7 (3+4) | B/B |
| 5 | 51 | pT2c | 7 (3+4) | B/B |
| 6 | 62 | pT2c | n.a. | B/B |
| 7 | 62 | pT2c | 7 (4+3) | B/B |
| 8 | 66 | pT3a | 7 (3+4) | B/B |
| 9 | 63 | pT2c | 7 (4+3) | B/B |
| 10 | 69 | pT2c | n.a. | B/B |
| 11 | 66 | pT3a | 7 (4+3) | B/B |
| 12 | 56 | pT2c | 6 (3+3) | B/B |
| 13 | 70 | pT3a | 7 (3+4) | B/B |
| 14 | 65 | pT2c | 5 (2+3) | B/B |
| 15 | 63 | pT2c | 6 (3+3) | B/B |
| 16 | 54 | pT2c | 7 (4+3) | B/B |
| 17 | 70 | pT2c | 7 (3+4) | B/B |
| 18 | 65 | pT2c | 7 (4+3) | B/B |
| 19 | 47 | pT3b | 8 (4+4) | B/B |
| 20 | 64 | pT3b | 8 (4+4) | B/B |
| 21 | 66 | pT2a | 6 (3+3) | A/B |
| 22 | 71 | pT2c | 9 (4+5) | B/B |
| 23 | 67 | pT3a | n.a. | B/B |
| 24 | 76 | pT2a | 7 (3+4) | B/B |
| 25 | 74 | pT3b | n.a. | B/B |
| 26 | 65 | pT3a | n.a. | B/B |
| 27 | 64 | pT3b | 10(5+5) | B/B |
| 28 | 61 | pT3a | 9 (5+4) | A/B |
| 29 | 54 | pT4 | 7 (3+4) | B/B |
| 30 | 74 | pT3a | 8 (4+4) | A/B |
| 31 | 72 | pT3b | 5 (2+3) | B/B |
| 32 | 74 | pT3b | n.a. | B/B |
| 33 | 65 | pT3b | 8 (4+4) | B/B |
| 34 | 68 | pT3a | 7 (3+4) | B/B |
| 35 | 67 | pT3a | 7 (3+4) | B/B |
| 36 | 66 | pT3a | 7 (4+3) | B/B |
| 37 | 52 | pT3b | 7 (4+3) | B/B |
| 38 | 59 | pT3a | 7 (4+3) | B/B |
| 39 | 69 | pT2c | 8 (3+5) | B/B |
| 40 | 61 | pT2c | 7 (3+4) | B/B |
| 42 | 66 | pT3b | 9 (4+5) | B/B |
| 43 | 65 | pT3b | 9 (4+5) | B/B |
| 44 | 63 | pT3a | 7 (3+4) | B/B |
| 45 | 62 | pT2a | 6 (3+3) | B/B |
| 46 | 68 | pT3a | 7 (4+3) | B/B |
| 48 | 70 | pT2c | 7 (3+4) | A/B |
| 49 | 59 | pT2a | 7 (3+4) | B/B |
| 50 | 69 | pT3a | 7 (3+4) | B/B |
| 51 | 59 | pT2c | 6 (3+3) | A/B |
| 52 | 59 | pT2c | 7 (3+4) | B/B |
| 53 | 62 | pT2c | 7 (3+4) | B/B |
| 54 | 66 | pT3a | 7 (4+3) | B/B |
| 55 | 66 | pT2a | 6 (3+3) | B/B |
| 56 | 75 | pT3a | 7 (3+4) | B/B |
| 57 | 62 | pT3a | 7 (4+3) | B/B |
| 58 | 66 | pT2c | 6 (3+3) | B/B |
| 59 | 69 | pT3a | 7 (3+4) | B/B |
| 60 | 67 | pT2a | 6 (3+3) | B/B |
| 61 | 70 | pT3a | 9 (4+5) | B/B |
| 62 | 65 | pT3a | 7 (4+3) | B/B |
| 63 | 52 | pT2c | 7 (3+4) | B/B |
| 64 | 60 | pT2c | 7 (3+4) | B/B |
| 65 | 61 | pT2c | 7 (3+4) | A/B |
| 66 | 60 | pT3a | 7 (3+4) | B/B |
| 67 | 71 | pT2a | 6 (3+3) | B/B |
| 68 | 57 | pT3b | 7 (4+3) | B/B |
| 69 | 73 | pT2c | 7 (4+3) | B/B |
| 70 | 61 | pT3a | 7 (4+3) | B/B |
| 71 | 62 | pT3a | 7 (4+3) | B/B |
| 72 | 63 | pT2 | 7 (3+4) | B/B |
| 73 | 60 | pT3a | 7 (4+3) | B/B |
| 74 | 72 | pT2c | 6 (3+3) | A/B |
| 75 | 67 | pT2c | n.a. | B/B |
| 76 | 56 | pT3a | 7 (3+4) | B/B |
| 77 | 73 | pT3b | 9 (4+5) | B/B |
| 78 | 57 | pT3a | 7 (4+3) | B/B |
| 79 | 70 | pT3a | 7 (4+3) | A/B |
| 80 | 74 | pT3b | n.a. | A/B |
| 81 | 57 | pT3a | 7 (3+4) | B/B |
| 82 | 61 | pT3b | 9 (5+4) | A/B |
| 83 | 71 | pT2c | 7 (4+3) | B/B |
| 84 | 75 | pT3b | 9 (4+5) | B/B |
| 85 | 72 | pT3b | 7 (4+3) | B/B |
| 86 | 61 | pT2c | 6 (3+3) | B/B |
| 87 | 62 | pT3b | 9 (4+5) | A/B |
| 88 | 73 | pT2c | 7 (3+4) | A/A |
| 89 | 70 | pT3a | 8 (4+4) | B/B |
| 90 | 65 | pT2c | 6 (3+3) | B/B |
| 91 | 71 | pT2c | n.a. | B/B |
| 92 | 59 | pT3b | 8 (4+4) | B/B |
| 93 | 63 | pT2c | 7 (3+4) | B/B |
| 94 | 58 | pT2c | 6 (3+3) | B/B |
| 95 | 67 | pT2c | 6 (3+3) | B/B |
| 96 | 67 | pT3a | 7 (3+4) | B/B |
| 97 | 66 | pT2c | 7 (4+3) | B/B |
| 98 | 70 | pT2a | 6 (3+3) | B/B |
| 99 | 63 | pT3b | 9 (5+4) | B/B |
| 100 | 61 | pT2c | 7 (4+3) | B/B |
| 101 | 73 | pT2c | 7 (3+4) | B/B |
| 102 | 71 | pT3a | 8 (5+3) | B/B |
